# The 2024 challenges in structural biology summit

**DOI:** 10.1063/4.0000752

**Published:** 2025-07-22

**Authors:** Brent L. Nannenga, Tamir Gonen

**Affiliations:** 1 Chemical Engineering, School for Engineering of Matter, Transport, and Energy, Arizona State University, Tempe, Arizona 85287, USA; 2 Center for Applied Structural Discovery, The Biodesign Institute, Arizona State University, Tempe, Arizona 85287, USA; 3 Howard Hughes Medical Institute, University of California, Los Angeles, Los Angeles, California 90095, USA; 4 Departments of Biological Chemistry and Physiology, David Geffen School of Medicine, University of California, Los Angeles, Los Angeles, California 90095, USA

## Abstract

In October 2024, the Challenges in Structural Biology Summit was held at the UCLA Lake Arrowhead Lodge. The meeting focused on new advancements and methods developments in structural biology. Here, we briefly summarize the 2024 Challenges in Structural Biology Summit.

The 2024 Challenges in Structural Biology Summit was held from October 15th to 18th at the UCLA Lake Arrowhead Lodge in Lake Arrowhead, California. As with previous meetings, the summit brought together method developers and researchers applying cutting edge methods in structural biology. While many meetings focus on the applications of these methods, this summit is unique in that the focus is on highlighting the methodology and discussing the next key areas of advancement to overcome various challenges in structural science. The meeting was attended by experts and developers in x-ray crystallography, single particle cryo-electron microscopy (cryo-EM), cryo-electron tomography (cryo-ET), and microcrystal electron diffraction (MicroED), and this year there were more attendees with expertise in NMR and computational protein structure prediction and analysis.

The presentations and discussions spanned the broad range of expertise at the meeting; however, there were a few key areas that were of particular interest at this year's meeting. High-throughput approaches were a common theme, with enhanced hardware and computational methodologies for x-ray crystallography, tomography, single particle, and electron diffraction all being discussed. There were also many presentations and discussions on the role of computational structure prediction tools in the future of structural biology. There were several talks on advanced structure prediction approaches, and many examples of how experimental structure determination can be supported and enhanced by these new structure prediction tools. Discussions on the future role of structure prediction and experimental structure determination also highlighted that the structural biology community has an obligation to educate the community about the best practices surrounding the use of protein prediction. Additionally, there were many lively discussions on the importance of educating and training the next generation of structural biologists as well as the benefits of open access science for the scientific community.

The need for the continued support and expansion of strong national structural biology user facilities was another important topic during the meeting. For the structural science user community to have access to the high-end instrumentation and expertise required for many of these methods, there needs to be increased support for facilities that enable user community access. This includes the existing synchrotron and XFEL facilities, NMR facilities, and cryo-EM national centers. Both synchrotron and single particle facilities would benefit from improvements to throughput to support the growing number of researchers using the facilities. The relatively new cryo-ET centers would benefit from continued investment to add instrumentation, which would enable more training and access to the technologies. Finally, it was suggested by the community that the creation of MicroED specific national facilities would greatly improve the access of the technique and facilitate new science.

**Figure:**
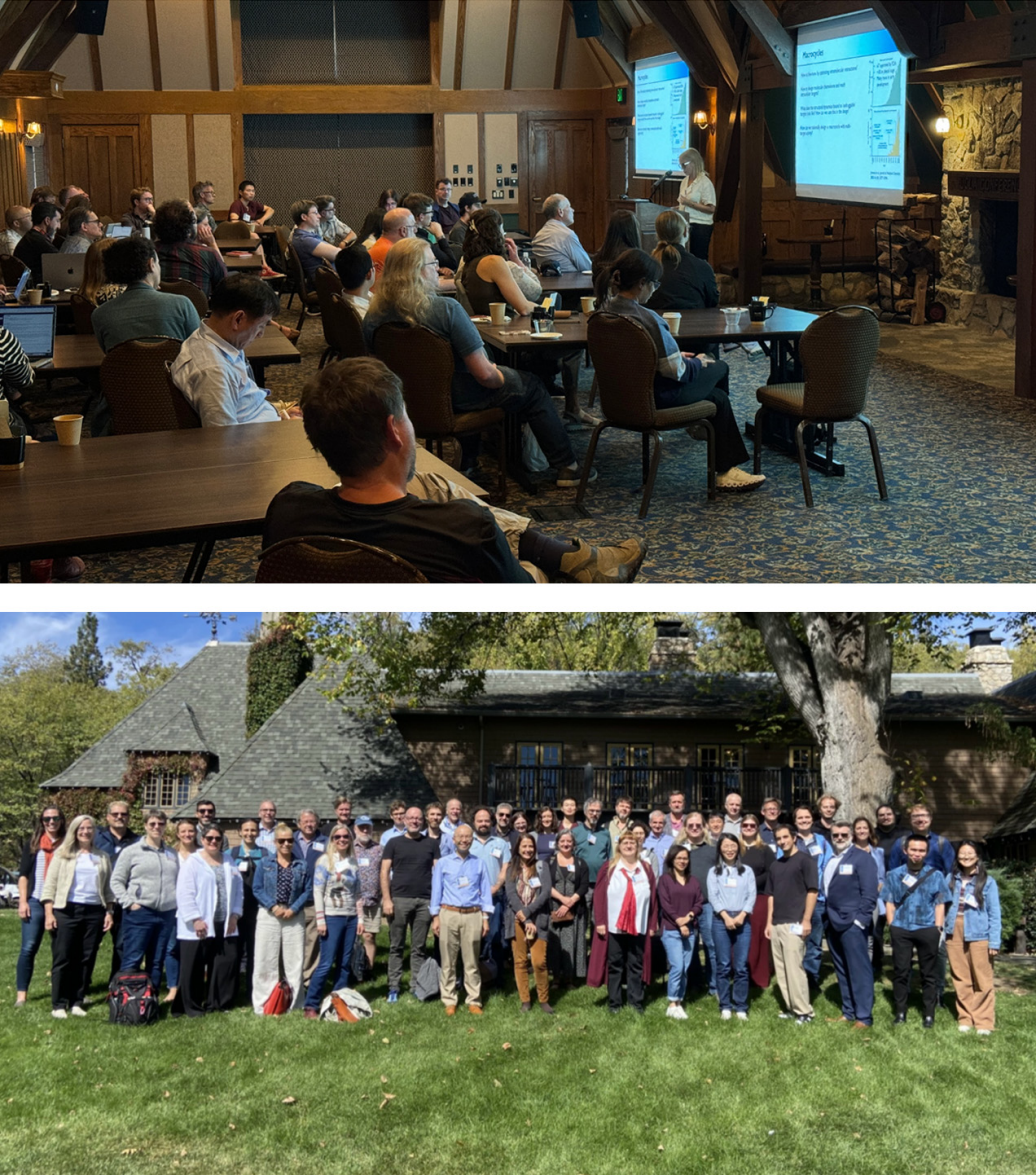


Overall, the 2024 Challenges in Structural Biology Summit was an exciting meeting that fostered interactions between researchers in structural methods development. We hope to continue to serve this vibrant community through future meetings that encourage continued advancements in new methodology and help to identify and overcome the challenges in the field. As a result of the summit, this special issue has been put together with submissions from several of the attendees. In the special issue, there are six articles describing various topics that were covered during the 2024 summit. Discussions on the integration of biophysical and structural approaches and how this integration is the future of structural biology were a central theme of the summit. In articles by Eng and Valdez and Eisenbaum *et al.*, tools and strategies for integrating structural data with other biophysical and computational approaches are discussed. A review by Ruma *et al.* covers the recent advances and approaches in cryo-EM. In Cuthbert *et al.*, the role of advanced glycation end products is described as well as evidence that these modifications are more common in protein structures than is currently appreciated in the field. The use of energy-filtering in MicroED data collection is presented by Clabbers and Gonen, which shows how electron diffraction data are significantly improved through the use of an energy filter. Finally, in work presented by Bu *et al.*, data obtained from crystal structures, solution structure, and docking studies are integrated to gain knowledge on how macrocyclic compounds bind their target molecules.

The 2024 structural biology summit was organized by Brent Nannenga and Tamir Gonen with the MicroED Imaging Center (MEDIC), which is supported by the NIH (Grant No. P41GM136508). The summit received generous support from Thermo Fisher Scientific, Rigaku, Quantum Detectors, and Gatan.

2024 Challenges in Structural Biology Summit. Attendees were Pavel Afonine, Michele Avissar-Whiting, Dominka Borek, Sarah Bowman, Aaron Brewster, Axel Brunger, Guillermo Calero, Yifan Cheng, Michael Cianfrocco, Max Clabbers, Emma Danelius, Radostin Danev, Roland Dunbrack, Edward Eng, Ignacia Echeverria, Megan Filbin, Paula da Fonseca, James Fraser, Petra Fromme, Marc Gallenito, Shane Gonen, Tamir Gonen, Celia Goulding, Danielle Grotjahn, Johan Hattne, Chi-Min Ho, Lisa Keefe, Masahide Kikkawa, Sven Klumpe, Rosalie Lawrence, Angeline Lyon, Alex de Marco, Rachel Martin, Michael Martynowycz, Brandon Mercado, Leonard Mueller, Brent Nannenga, Zbyszek Otwinowski, Sarah Perry, George Phillips, Lauren Porter, Stefan Raunser, Nicholas Sauter, Piotr Sliz, and Edward Twomey.

## Data Availability

Data sharing is not applicable to this article as no new data were created or analyzed in this study.

